# Path and Ridge Regression Analysis of Seed Yield and Seed Yield Components of Russian Wildrye (*Psathyrostachys juncea* Nevski) under Field Conditions

**DOI:** 10.1371/journal.pone.0018245

**Published:** 2011-04-18

**Authors:** Quanzhen Wang, Tiejun Zhang, Jian Cui, Xianguo Wang, He Zhou, Jianguo Han, René Gislum

**Affiliations:** 1 Department of Grassland Science, College of Science and Technology, Northwest A&F University, Yangling, Shaanxi Province, People's Republic of China; 2 Institute of Grassland Science, College of Animal Science and Technology, China Agricultural University, Beijing, People's Republic of China; 3 Institute of Animal Science, Chinese Academy of Agricultural Sciences, Beijing, People's Republic of China; 4 Department of Genetics and Biotechnology, Faculty of Agricultural Science, Aarhus University, Slagelse, Denmark; University College London, United Kingdom

## Abstract

The correlations among seed yield components, and their direct and indirect effects on the seed yield (Z) of Russina wildrye (*Psathyrostachys juncea* Nevski) were investigated. The seed yield components: fertile tillers m^-2^ (Y_1_), spikelets per fertile tillers (Y_2_), florets per spikelet^-^ (Y_3_), seed numbers per spikelet (Y_4_) and seed weight (Y_5_) were counted and the Z were determined in field experiments from 2003 to 2006 via big sample size. Y_1_ was the most important seed yield component describing the Z and Y_2_ was the least. The total direct effects of the Y_1_, Y_3_ and Y_5_ to the Z were positive while Y_4_ and Y_2_ were weakly negative. The total effects (directs plus indirects) of the components were positively contributed to the Z by path analyses. The seed yield components Y_1_, Y_2_, Y_4_ and Y_5_ were significantly (P<0.001) correlated with the Z for 4 years totally, while in the individual years, Y_2_ were not significant correlated with Y_3_, Y_4_ and Y_5_ by Peason correlation analyses in the five components in the plant seed production. Therefore, selection for high seed yield through direct selection for large Y_1_, Y_2_ and Y_3_ would be effective for breeding programs in grasses. Furthermore, it is the most important that, via ridge regression, a steady algorithm model between Z and the five yield components was founded, which can be closely estimated the seed yield via the components.

## Introduction

Forages are the backbone of sustainable agriculture and environmental regeneration in arid land [1]. Perennial forage crops play a major role in providing high quality feed for the economical production of meat, milk and fiber products [2]. Perennial forage crops are also important in soil conservation and environmental protection [3], as they add organic matter to the soil and serve as a permanent ground cover preventing soil erosion [4]. In addition, perennial grasses are potentially useful for crop improvement as they possess important germplasm or genes for being tolerant to rigorous environment (field conditions) [5], [6].

Russian wildrye (*Psathyrostachys juncea* Nevski) is a perennial grass, which is growing rapidly, highly drought and CaCO_3_ tolerant and has a low fertility requirement [7], [8], [9], [10]. Russian wildrye is a cool-season forage species well adapted to semi-arid climates [3], [11]. It is a perennial bunchgrass and is characterized by dense basal leaves that retain their nutritive value better during the late summer and autumn than many other grasses [12].

Established stands of Russian wildrye provide excellent grazing for livestock and wildlife on semi-arid rangelands of the Intermountain West and the Northern Great Plains in North America [3], [13], [14]. Also, it is very competitive, high-yielding, an excellent source of forage for livestock and wildlife on semi-arid rangelands [12] in Eurasia and northwest China [4], [9], [10], [11], [15], [16], and it is also an important forage crop for revegetating rangeland in North America [17]and northwest China [1], [9]. In addition, Russian wildrye is cross-pollinated and relatively self-sterile [14]. It is the only agriculturally important species in the genus *Psathyrostachy*s, which is a member of the *Triticeae* tribe [16], [18] and is also considered to be an important germplasm in crop improvement as it possesses resistance to barley yellow dwarf virus (BYDV) [1], [3], [10], [19].

There is a limited use of Russian wildrye due to its unsteadiness of seed production [1]. The reason is most probably that breeding programs has focused on developing Russian wildrys cultivars with a high biomass yield while improvement of seed yield has been neglected. Seed yield is a quantitative character, which is largely influenced by the environment and hence has a low heritability [20]. Therefore, the response to direct selection for seed yield may be unpredictable, unless there is good control of environmental variation. In order to select for higher seed yield there is the need to examine the mathematical relationships among various characters, especially between seed yield and key seed yield components and a certain amount of interdependence between them [21], e.g. seed yield components do not only directly affect the seed yield, but also indirectly by affecting other yield components in negative or positive ways [22]. In such situations, knowledge of the nature of genetic variability and interrelationships among seed yield and key yield components would facilitate with reference to breeding improvement for these traits [23]. Another possibility would be: To unravel the often complicated interdependence between seed yield components and seed yield knowledge of the nature on genetic variability and interrelationships among seed yield and seed yield components is important. This knowledge also merits future breeding programs in Russian wildrye. To our knowledge no information is available on the mathematical relationship between seed yield and seed yield components in Russian wildrye.

Path analysis provides a method of separating direct and indirect effects and measuring the relative importance of the causal factors involved. Several researchers have used this method to assess the importance of the components of yield [20], [23], [24], [25]. The advantage of path analysis is that it permits the partitioning of the correlation coefficient into its components, one component being the path coefficient that measures the direct effect of a predictor variable upon its response variable; the second component being the indirect effect(s) of a predictor variable on the response variable through another predictor variable [26]. In agriculture, path analysis has been used by plant breeders to assist in identifying traits that are useful as selection criteria to improve crop yield [26], [27].

For grass crops, the correlation of economic yield components with seed yield and the partitioning of the correlation coefficient into its components of direct and indirect effects have been extensively reported: e.g. highly significant associations of grain yield were observed with 1000-grain weight and tiller number per plant [28], [29], the number of filled grains per panicle and harvest index [30]. Grain yield has been influenced by high direct effects of total tillers and days to flowering [31], the number of panicles per plant, the number filled grains per panicle and 1000-grain weight, the number of filled grains per panicle and plant height, productive tillers, panicle length and flowering time [21], [32], plant height and tiller number, panicle number per plant, spikelet number per panicle, the number of effective tillers per plant, grains per panicle and 1000-grain weight, grains per panicle and productive tillers [33], the number of filled grains per panicle and 1000-grains weight [34] and biological yield, harvest index and 1000-grain weight, etc., but few of about grass seed yield components. Such detailed cause and effect mathematical relationships have not been examined in *Psathyrostachys juncea* Nevski.

However, morphological characters influencing yield are often highly inter-correlated, leading to multi-collinearity when the inter-correlated variables are regressed against seed yield in a multiple-regression equation. For such situations estimation of regression coefficients through ridge-regression was developed by Hoerl and Kennard [35] to ameliorate problems like inflation in absolute value of the regression coefficients and wrong sign of the regression coefficients resulting from these inter-correlated variables.

Based on multi-factor orthogonal design of various field experimental management, with big sample size, the main objective of this study was to examine the mathematical relationships between the seed yield (Z) and the key seed yield components: fertile tillers m^-2^ (Y_1_), spikelets per fertile tillers (Y_2_), florets per spikelet (Y_3_), seed numbers per spikelet (Y_4_) and seed weight (mg) (Y_5_) in Russian wildrye. Then there are formulas theoretically. Seed yield:
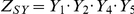



If one floret equals one seed embryo for grasses, then, Seed yield potential:
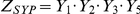



The mathematical relationship was examined using path coefficient and ridge regression analysis. Our hypothesis was that: 1) all the five seed yield components and the seed yield are inter-correlated, and all the five seed yield components are positively contributed to seed yield and 2) the relationship between seed yield and the five seed yield components should be a steady algorithm model which can be closely estimated the seed yield via the components.

## Results

Pearson correlation coefficients for all the four years totally shows that seed yield components Y_1_, Y_2_ and Y_4_ are significantly (P<0.0001) positive correlated with the Z, while Y_5_ is significantly (P<0.01) negative correlated with the Z (Table 1). There was a negative significant correlation between Y_1_ and Y_3_ and between Y_1_ and Y_5_, while the correlation between Y_2_ and Y_5_ was non-significant it was still negative. The Pearson correlation of the Z and its components for individual years analyses of 2003, 2004, 2005 and 2006 showed that only Y_1_ in all the four years are positively significant correlated with Z and Y_2_ (P≤0.01), the correlation coefficients of the years order is: 2004>2003>2006>2005 and 2006>2004>2005>2003, respectively (Table 2). The Y_3_ with Y_4_ exhibited positively significant correlation in 2003, 2004 and 2005 along with the Y_1_ with Y_3_ in 2004, 2005 and 2006 and Y_2_ with Z in 2004, 2005 and 2003. The Y_3_ and Y_4_ with Y_5_ exhibited positively significant correlation in 2004 and 2005 (P<0.0001) (Table 2).

**Table 1:** Pearson correlation coefficients of Y_1_∼Y_5_, Z (*Psathyrostachys juncea* Nevski) for 4 years totally.

Seed yield components	Y_1_	Y_2_	Y_3_	Y_4_	Y_5_	Z(seed yield)
Y_1_	1.0000	0.4920***	-0.3535***	0.2002***	-0.3600***	0.8182***
Y_2_		1.0000	0.2012***	0.2893***	-0.0775	0.4554***
Y_3_			1.0000	0.5866***	0.4226***	-0.0781
Y_4_				1.0000	0.1865***	0.3570***
Y_5_					1.0000	-0.1745**
Total sample size (n)	3150	10080	9135	11970	3150	1260

F-values are presented along with statistical differences:

*P<0.05,

**P<0.01,

***P<0.0001. N = 315

**Table 2:** Pearson correlation coefficients of Y_1_∼Y_5_, Z (*Psathyrostachys juncea* Nevski) for each year.

	year	Y_1_	Y_2_	Y_3_	Y_4_	Y_5_	Z
Y_1_	2003	1.0000	0.3091**	0.1067	0.1317	-0.0081	0.7494***
	2004	1.0000	0.5973***	0.2101*	0.2428**	-0.0122	0.8045***
	2005	1.0000	0.5312***	-0.4456**	-0.2632*	-0.5762***	0.3985**
	2006	1.0000	0.6430**	-0.5561*	-0.0450	0.0269	0.6245**
Y_2_	2003		1.0000	-0.0712	-0.1283	-0.0217	0.1954*
	2004		1.0000	-0.1610	-0.0160	-0.1953*	0.3783***
	2005		1.0000	-0.1024	0.1305	-0.1588	0.3165*
	2006		1.0000	-0.1111	0.1062	-0.0717	0.4036
Y_3_	2003			1.0000	0.9276***	0.1588	0.1276
	2004			1.0000	0.7087***	0.3291***	0.3420***
	2005			1.0000	0.6443***	0.6295***	-0.0394
	2006			1.0000	0.4531	0.1794	0.0271
Y_4_	2003				1.0000	0.1223	0.1106
	2004				1.0000	0.3210***	0.3121***
	2005				1.0000	0.5634***	0.0290
	2006				1.0000	-0.0519	0.2654
Y_5_	2003					1.0000	0.2320*
	2004					1.0000	-0.0257
	2005					1.0000	-0.979
	2006					1.0000	0.4398

F-values are presented along with statistical differences:

*P<0.05,

**P<0.01,

***P<0.0001. N = 105, 134, 60 and 16 for year 2003, 2004, 2005 and 2006, respectively.

Direct and indirect effects of Y_1_∼Y_5_ on the seed yield are presented in Table 3. In the individual years from 2003 to 2006 all five seed yield components had a significantly correlated relationship with Z in at least one year (Table 2), however, path analysis showed that only Y_1_ had strong direct effect (highlighted in bold in Table 3) on Z in the total 4 years (2003 and 2004 are at P≤0.0001, 2005 and 2006 are at P≤0.05), the coefficients are 0.7741, 0.8268, 0.4568 and 0.9417 respectively, thus Y_1_ had largest contribution to Z among them. And, Y_5_ in 2003 (0.2309 at P≤0.0001) and Y_3_ in 2004 (0.1672 at P≤0.05) significantly had direct effect on Z. Furthermore, via SAS, the results of ridge regression analysis and Duncan's Multiple Range Test for seed yield (z) and its components (Y_1_∼Y_5_) of the 4 years are showed in Table 4.

**Table 3:** Path analysis showing direct and indirect effect of Y_1_∼Y_5_ to Z (*Psathyrostachys juncea* Nevski).

	year	Indirect effect via				
		→Y_1_→Z	→Y_2_→Z	→Y_3_→Z	→Y_4_→Z	→Y_5_→Z
Y_1_	2003	**0.7741** ***	0.0604	0.0136	0.0146	-0.0019
	2004	**0.8268** ***	0.2260	0.0719	0.0758	0.0003
	2005	**0.4568** *	0.1681	0.0175	-0.0076	0.0564
	2006	**0.9417** *	0.2595	-0.0150	-0.0119	0.0118
Y_2_	2003	0.2317	**-0.0522**	-0.0091	-0.0142	-0.0050
	2004	0.4805	**-0.1076**	-0.0551	-0.0050	0.0051
	2005	0.2117	**0.1009**	0.0040	0.0038	0.0155
	2006	0.4015	**-0.1500**	-0.0030	0.0282	-0.0315
Y_3_	2003	0.0799	-0.0139	**0.2082**	0.1025	0.0368
	2004	0.1691	-0.0609	**0.1672** *	0.2212	-0.0085
	2005	-0.1776	-0.0324	**0.0956**	0.0187	-0.0616
	2006	-0.3473	-0.0448	**0.4007**	0.1202	0.0789
Y_4_	2003	0.0987	-0.0251	0.1183	**-0.2195**	0.0284
	2004	0.1953	-0.0061	0.2424	**0.0229**	-0.0082
	2005	-0.1049	0.0413	-0.0254	**0.0090**	-0.0552
	2006	-0.0281	0.0429	0.0123	**0.1597**	-0.0228
Y_5_	2003	-0.0061	-0.0042	0.0202	0.0135	**0.2309** ***
	2004	-0.0098	-0.0739	0.1125	0.1002	**-0.0990**
	2005	-0.2300	-0.0502	-0.0248	0.0163	**0.1161**
	2006	0.0168	-0.0289	0.0049	-0.0138	**0.3401**
Total direct effect		**2.9994**	**-0.2089**	**0.8717**	**-0.0279**	**0.5881**
Total effect		3.9808	0.2489	1.3569	0.6346	0.6266

F-values are presented along with statistical differences:

*P <0.05,

**P <0.01,

***P <0.0001.

The direct effects of Y_1_∼Y_5_ to z are highlighted in bold (on main diagonal cell); Arrows illustrate directions of effects. pye = 0.6117, 0.5556, 0.8949 and 0.5192 for year 2003, 2004, 2005 and 2006, respectively.

**Table 4:** Duncan's Multiple Range Test for seed yield (z) and its components (Y_1_∼Y_5_) of *Psathyrostachys juncea* Nevski of the 4 years, and of the ridge regression coefficients.

	year	N	Y_1_	Y_2_	Y_3_	Y_4_	Y_5_	Z
Duncan's Multiple Range Test								
	2003	105	205.67 c	90.22 a	4.590 a	2.141 a	3.461 a	964.4 b
	2004	134	542.31 a	89.54 a	2.358 b	2.054 a	3.093 b	1483.8 a
	2005	60	178.09 c	82.34 b	2.293 b	1.587 c	3.387 a	541.3 c
	2006	16	338.47 b	81.14 b	2.231 b	1.749 b	2.856 c	714.4 c
	F Value		89.35	31.93	548.55	70.62	39.34	55.35
	Pr > F		<.0001	<.0001	<.0001	<.0001	<.0001	<.0001
Ridge regression coefficients								
k	year	Intercept	Y_1_	Y_2_	Y_3_	Y_4_	Y_5_	Z
0.6	2003	-892.634	2.188	4.607	15.461	3.201	263.961	-1
0.6	2004	-1611.481	1.164	7.456	510.828	274.322	7.807	-1
0.7	2005	-423.256	0.651	8.670	31.712	33.030	2.848	-1
0.6	2006	-827.011	0.667	5.076	73.065	159.624	161.698	-1

Means with the same letter are not significantly different at Alpha = 0.05.

As for the contributions of Y_1_ to Y_5_ to Z, viewing totally the result of each 4 year as a group, the strongest indirect effect toward Z is Y_2_ via Y_1_ (the coefficients are 0.2317, 0.4805, 0.2117 and 0.4015), then orderly come Y_1_ via Y_2_ (0.0604, 0.2260, 0.1681 and 0.2595) and Y_3_ via Y_4_ (0.1025, 0.2212, 0.0187 and 0.1202). Y_5_ via Y_2_ had lightly a negative indirect effect to Z (-0.0042, -0.0739, -0.0502 and -0.0289). Combining the direct effects (highlighted in bold) of Y_2_ to Z had negative effects in 3 years (2003, 2004 and 2006) and positive effect in 1 year (2005), obviously, Y_2_ had least contribution to Z.

Y_3_ had positive effects to Z in four years, whereas Y_4_ and Y_5_ had a negative effect in one year respectively. In addition, Y_5_ had more contribution to Z than Y_4_ by comparing the coefficients between them from Table 3.

So, The contributions of the five seed yield components to the seed yield are orderly Y_1_>Y_3_>Y_5_>Y_4_>Y_2_. The order is the same as total direct effects (2.9994, -0.2089, 0.8717, -0.0279 and 0.5881 listed in Table 3) with Y_4_ and Y_2_ having negative effects, but the total effects order is Y_1_>Y_3_>Y_4_>Y_5_>Y_2_ (3.9808, 0.2489, 1.3569, 0.6346 and 0.6266 listed in Table 3).

Duncan's Multiple Range Test for seed yield (Z) and its components (Y_1_ to Y_5_) Showed that Z was significantly highest in 2004 followed by 2003 which was significant higher than 2005 and 2006 (Table 4). Y_1_ was the highest in 2004 and produced the highest Z. Except in 2003, Y_3_ was not significantly (P<0.05) different in the rest three years.

The ridge regression and multiple-regression was applied for avoiding the highly inter-correlated and multi-collinearity between Y_1_ to Y_5_ and Z [35], [36], [37],[38],[39].

**Figure 1:**
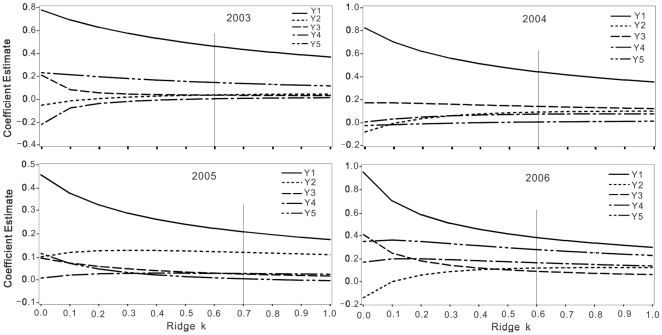
Ridge traces of standard partial regression coefficients for increasing values of k for five yield components for year 2003, 2004, 2005 and 2006 respectively. Y_1_ to Y_5_ are stand for fertile tillers m^-2^, spikelets per fertile tillers, florets per spikelet, seed numbers per spikelet and seed weight, respectively.



There are several procedures have been proposed for the selection of k in ridge regression analysis, although the optimal value of k cannot be determined with certainty [36], [37], [39], [40], and suggested that k should be determined from the ridge trace, with k selected such that a stable set of regression coefficients was obtained [38]. In this study, Figure 1 for year 2003, 2004, 2005 and 2006 respectively, showed the standard ridge traces, for various values of k, viewing the curves of Y_1_ to Y_5_ were asymptotically parallel to the horizontal axis when with the values of k estimated at the point 0.6, 0.6, 0.7 and 0.6 respectively, using the method of Horl and Kennard [35], [36], the ridge regression models were obtained at the selected values of the k for year 2003, 2004, 2005 and 2006, respectively. The resulting ridge regression coefficients are shown in Table 4. The ridge regression models were A, B, C and D, for year of 2003, 2004, 2005 and 2006, respectively:

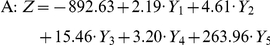



(Ridge k  = 0.6; F  = 33.11 Pr<0.0001)
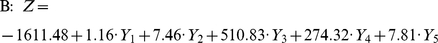



(Ridge k  = 0.6; F  = 57.33 Pr<0.0001)
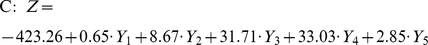



(Ridge k  = 0.7; F  = 2.68 Pr<0.0308)
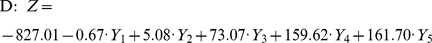



(Ridge k  = 0.6; F  = 5.42 Pr<0.0114)

All of the ridge coefficients were positive whereas the values were various in the 4 years (Table 4). The highest ridge regression coefficients of Y_1_ and Y_5_, Y_3_ and Y_4_, and Y_2_ were in 2003, 2004, and in 2005 respectively (Table 4). Partly due to sample size, the ridge models in 2005 and 2006 was significant at Pr<0.05.

All of the Z and Y_1_ to Y_5_, 315 samples from the database of the 4 years totally, were taken the natural logarithm as S and C_1_ to C_5_, then S and C_1_ to C_5_ were taken in for ridge regression analyses, and got ridge regression model as:
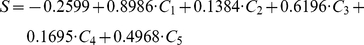
(1)


(N = 315, F = 142.34, Pr<.0001)

Thus,
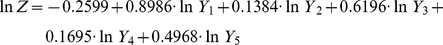



Above logarithmic model was transformed to exponential function as:
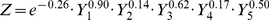
(2)


Formula (2) was used to estimate the seed yield of all the 315 samples and denoted as Z_estimated_. The actual seed yields were denoted as Z_actual_.

**Figure 2:**
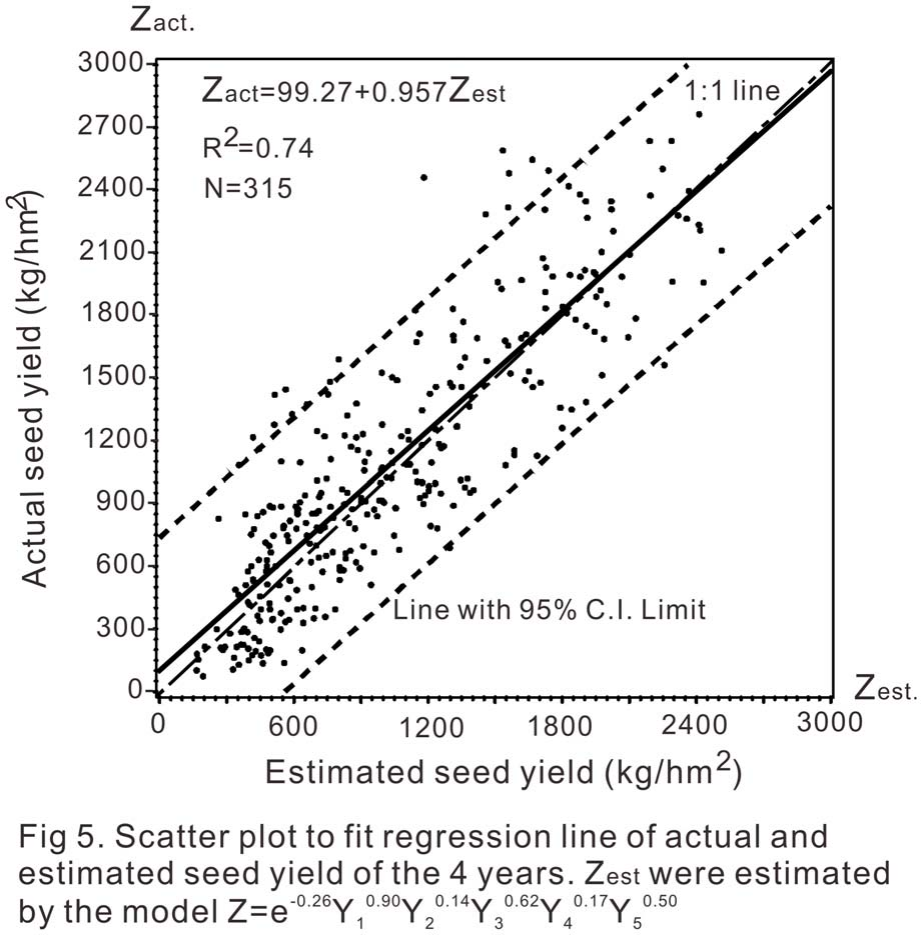
Scatter plot to fit regression line of actual and estimated seed yield of the 4 years. Zest were estimated by the model Z = e^-0.26^ *Y*_1_^0.90^ *Y*_2_^0.14^ *Y*_3_^0.62^ *Y*_4_^0.17^ *Y*_5_^0.50^.

Then a general linear regression model was used to assess the Z_actual_ as compared to the Z_estimated_. And analysis of variance for dependent variable Z_actual_ and the parameter estimates of Z_estimated_ was showed in Table 5 and 6. The linear line was presented in Figure 2 with the regression model as:

**Table 5:** Analysis of variance for dependent variable Z_actual_.

Source	DF	Sum of squares	Mean square	F value	Pr > F
Model	1	93271881	93271881	896.67	<.0001
Error	313	32558436	104021		
Corrected total	314	125830318			

**Table 6:** Parameter estimates of Z_estimated_.

Variable	DF	Parameter estimate	Standard error	t value	Pr > |t|
Intercept	1	99.27080	37.71898	2.63	0.0089
Z_estimated_	1	0.95699	0.03196	29.94	<.0001



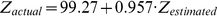
(3)(N = 315, F = 896.67, Pr<.0001)

So, via formula (3), the model was adjusted as:
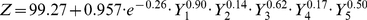
(4)


**Figure 3:**
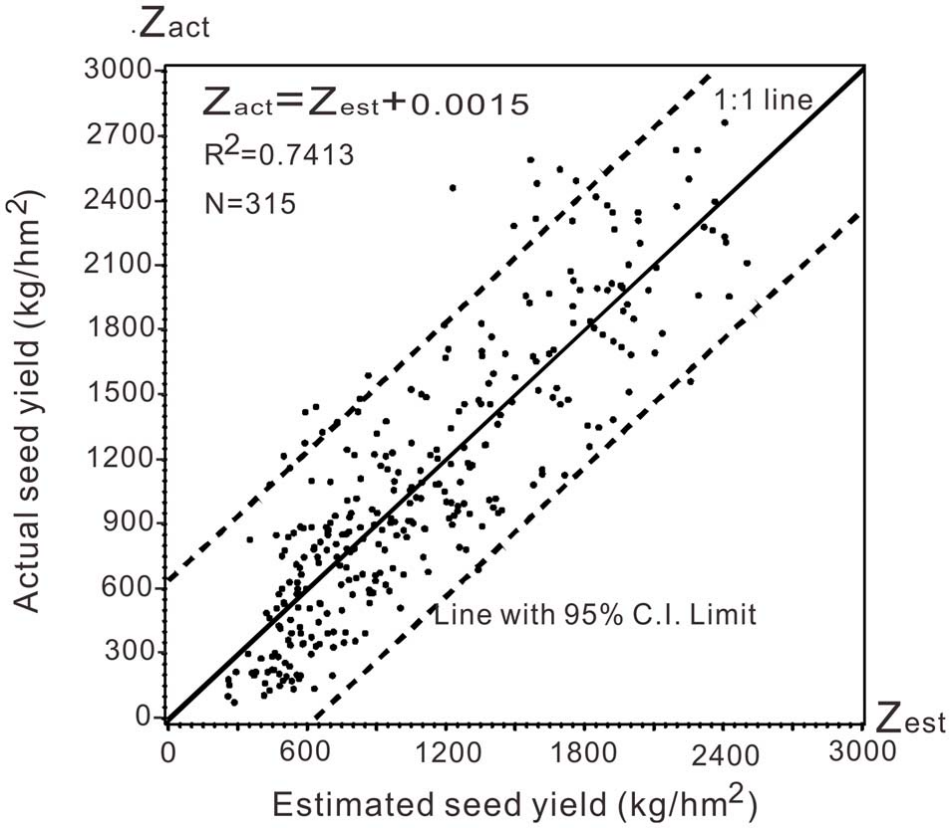
Scatter plot to fit regression line of actual and estimated seed yield adjusted by Z_act_ = 99.27+0.957·*Z*_est_ of the 4 years. It is superposed on the 1∶1 line.

By variance test, the parameter estimates of intercept and Z_estimated_ were 0.00153 and 0.99999 respectively (showed in Table 7). And the linear line, presented in Figure 3, was superposed on the 1:1 line.

**Table 7:** Parameter estimates of Z_estimated_ after adjusted by the linear regression.

Variable	DF	Parameter estimate	Standard error	t value	Pr > |t|
Intercept	1	0.00153	40.65539	0.00	1.0000
Z_estimated_	1	0.99999	0.03339	29.94	<.0001

## Discussion

The results suggest that our first hypothesis that Y_1_ to Y_5_ and the Z are inter-correlated, and all the five key seed yield components are positively contributed to Z could not be validated. However, our second hypothesis that a steady algorithm model, which can estimate the seed yield via the components, was found.

### Seed yield components and seed yield

Results show that total direct effects of Y_1_, Y_3_ and Y_5_ were positively contributed to Z but Y_4_ and Y_2_ were negatively; whereas the total effects (indirect + direct) of Y_1_∼Y_5_ to Z are positive. The negative effects of Y_2_ and Y_4_ were mainly canceled out by the effects of Y_1_ via Y_2_ (Y_1_→Y_2_) and Y_3_ via Y_4_ (Y_3_→Y_4_), respectively. There was no results available on negative effects of Y_2_ and Y_4_ in Russian wildrye. Firstly, Y_2_ is mostly genetic control [41], [42], there is not significantly different between 2003 and 2004 or between 2005 and 2006, and it decreases from 90.22 in 2003 to 81.14 in 2006 with increasing density because of aging (Table 4). Y_4_ has the same trend as Y_2_ with aging from 2.14 in 2003 to 1.75 in 2006. The large seed number (Y_4_) has a weak negative effect on seed yield maybe from the reason of limited soil nutrition with higher density [43]. Secondly, It maybe a true mathematical relationship resulting from a big sample size, e.g. both Y_2_ and Y_4_ are 4020 samples in 2004 in this research.

The seed yield component Y_1_ was the most important and effective component for seed yield, Z for significantly (P<0.0001 in 2003 and 2004; P<0.05 in 2005 and 2006) coefficients (0.7741, 0.8268, 0.4568 and 0.9417); this is in accordance with former experiments in Russian wildrye [44], [45], in fescues [46], [47], in zoysiagrass [48], in smooth brome [49], in perennial ryegrass [50] and in grasses [2], [51] and legumes [51], [52]. In addition, it was inferred that path-analysis could uncover the relationships between the components and the yield agreed with parallel results [53], [54], [55], [56]. As a seed yield component (Y_1_ to Y_5_) can affect other components positively or negatively, it is clear that measurement of simple linear relationships between two components with correlation analysis does not predict the success of selection. But, with standardized variables, path-analysis effectively determined the relative importance of direct and indirect effects on Z.

### Steady algorithm model to estimate Z via Y_1_ to Y_5_

An exponential model was founded for estimating the Z via Y_1_ to Y_5_. Firstly, it deduced from the data of 315 samples in variously growing management in successive 4 years elaborate with more words. Secondly, it was of the same order of exponent values in the model as that of the contributions of the five components to Z; this mean that there was much correspondence between path-coefficients analysis and the ridge regressions. Thirdly, all of the four ridge regression models of the individual years were significant (2003 and 2004 at P<0.0001; 2005 and 2006 at P<0.05), and all with positive coefficients (Table 4). In addition, with multi-factor orthogonal experimental designs and big sample statistical analysis in field experiment, the significant (at P = 0.0001 and 0.01) coefficients of the correlation, path analyses and ridge regressions show that the models are reliable, and that ridge regression effectively overcome the problem of highly multi-correlated predictor variables (Y_1_ to Y_5_) [35], [36]. This research method may be one of the efficient and effective method in field crop experiment [39], [57], [58]. Unfortunately, the coefficients of the ridge regression models in individual years were various, ranged from 0.651 to 510.83 (Table 4), maybe mainly due to aging of the plant, designed field management and various climates.

### Not all the five components and Z are inter-correlated

Though the experiment was set in various conditions with big sample size, the results of correlation analyses seems that theoretically accorded with biological theory in this experiment. Except Y_1_ with Y_2_ and Y_1_ with Z, the significant correlations were various. This was probably a consequence of the effects under climate of the individual year as the fields management are yearly repeats.

### The relationships of Z and Ys are highly associated with the climate

Due to designed various field experimental management (experimental factor X_1_ to X_10_), there was a very wide range of seed yield and its yield components (Table S2), for example, in 2004 the maximum seed yield is 2763.89 kg/hm^2^, and the minimum 74.64 kg/hm^2^ (due to low/no irrigation, no fertilization and few plants) this plot have got a few irrigation, no any fertilizing and with the least fertilized tillers and plants, in terms of average, *Psathyrostachys juncea* Nevski. Z and its yield components (Y_1_, Y_2_, Y_4_ and Y_5_) are very different between the years of 2003∼2006 (Table S2); besides aging of the plant, this is the main effect of weather conditions of the 4 years (Figure S1). For example, that there were higher rainfall in June, which was the seed growing period, in 2003 and 2004 than in 2005 and 2006 partly result in higher seed yields as it in favor of pollination and grain filling. The most rainfall was in March 2005 which also had lower air temperature facilitated vegetative growing and decreased Y_1_ (Table 4) and consequently resulted in a lower Z. In comparison, the highest Z matched the higher temperature in March and April in 2004 than in other years. However, Y_2_ and Y_3_ were weakly decreased going with aging of the plant from 2003 to 2006; they might be controlled by its genotypes in some degree in this experimental site.

### Conclusions

Via ridge regression analysis with big sample size in *Psathyrostachys juncea* Nevski, the model of seed yield with its five components was:
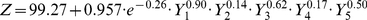
(5)


The total direct effects of the Y_1_, Y_3_ and Y_5_ to the seed yield were positive but Y_4_ and Y_2_ weakly negative; whereas the total effects (directs plus indirects) of the components were positively contributed to the seed yield by path analyses. Except Y_3_, Y_1_, Y_2_, Y_4_ and Y_5_ were significantly (P<0.001) correlated with the seed yield whereas Y_2_ were not significant correlated with Y_3_, Y_4_ and Y_5_ by Peason correlation analyses. Y_1_ was the major component presenting the most important and effective effect in the 5 components in the plant seed production. Therefore, selection for high seed yield through direct selection for large Y_1_, Y_2_ and Y_3_ would be effective for breeding programs in grasses.

The future study maybe consider the climate, e.g. rainfall and temperature in the seed growing stage, and different site locations for determining and testing the algorithm models of seed yield with the seed yield components in grasses.

## Materials and Methods

### Research Location and field conditions

Field experiments were conducted at the China Agricultural University Grassland Research Station located at the Hexi Corridor, in Jiuquan, Gansu province, northwestern China (latitude 39°37′N, longitude 98°30′E; elevation 1480 m) from 2003 to 2006. Soil at the site is Mot-Cal-Orthic Aridisols, classified as Xeric Haplocalcids (Soil Survey Staff, 1996). The 0.6 hm^2^ experimental site was tilled using a chisel plow in the fall and a disk-harrow in the spring for seedbed preparation. Russian wildrye (*Psathyrostachys juncea* Nevski) seeds (Cultivar: Bozoisky), were planted on 23 April 2002 at planting depth of 2.5 cm, a seeding rate of 5×10^6^ seeds hm^−2^ and a row distances of 0.45 m. The former crop was alfalfa (*Medicago sativa* L.). Nitrogen (pure N) in rates of 104 kg hm^−2^ and phosphorus in rates of 63 kg hm^−2^ P_2_O_5_ was applied in bands 6 cm deep and 5 cm to the side of seed furrow. There was no seed yield in autumn 2002. This research trial was carried on in the next four years (2003 to 2006) with designed field managements (x_1∼10_), at yearly repeat (Table S1).

### Experimental design

To simulate various growing conditions, the experiment used six groups (Group A to F) of multi-factor orthogonal field experimental designed plots [57], [59], [60], [61] (Table S1). Totally 143 experimental plots with different treatments combinations were arranged. Each one of individual plot areas 28 m^2^ (i.e. 4 m ×7 m), and with 1.5 m spacing between the adjacent plots. Weather for the experimental sites was provided by The Meteorological Working Station in Jiuquan, of Gansu province, P R China (Figure S1).

According to the orthogonal experimental designs, yearly repeated, under various field management, conditions from controlled growing environments, including regimes of fertilized (experimental factor: X_1_, X_3,_ and X_4_), irrigation system (experimental factor: X_2_), planted density (experimental factor: X_5_), spray plant regulators (experimental factor: X_6_), irrigation time (experimental factor: X_7_), density manipulation (experimental factor: X_8_), time of cut post-harvest stubbles (experimental factor: X_9_), and burning post-harvest stubbles (experimental factor: X_10_), are listed in Table S1.

### Data collection

Ten samples of 1 m length row were randomly selected for measuring the five seed yield components from anthesis to seed harvest during 2003 to 2006 respectively, for avoiding marginal utility, leave out 1 m from edge in the plots, which is means that samples were taken in the middle of the plot to avoid edge effect, the data of the seed yield components and seed yields of each one plot were collected by tactics as following: the samples of 1 m length row were randomly selected for measuring fertile tillers m^-2^ (Y_1_). Respectively, 30 to 36 fertile tillers and 27 to 54 spikelets were randomly selected for measuring the spikelets per fertile tillers (Y_2_), florets per spikelet (Y_3_) and seed numbers per spikelet (Y_4_). When the seed heads were ripen, four samples of 1 m length row were separately threshed by hand; yield of clean seed for each sample was weighted while the seed water content is at 7 to 10% for converting into seed yield (kg hm^-2^) (Z), and randomly taken 10 lots of 100-grains for determining seed weight (mg) (Y_5_) from the samples respectively. That total numbers of samples (n) of Y_1_ to Y_5_ and Z are 3150, 10080, 9135, 11970, 3150 and 1260 were determined respectively in the 4 years (Table 8). The sample size of been determined were listed in the individual years (Table 8), and then established experimental databases with Visio FoxPro (Version 6.0). Dates of flowering and seed harvesting in 2003 to 2006 (Table S3).

**Table 8:** The sample size of Y_1_∼Y_5_, z for each field experimental plot on *Psathyrostachys juncea* Nevski.

year	Sample size of plots (N) (treatment)	Sample size of each field experimental plot					
		Fertile tillers/m^2^Y_1_ (no.)	Spiklets/fertile tillersY_2_ (no.)	Florets/spikletY_3_ (no.)	Seed numbers/spikletY_4_ (no.)	Seed weight^a^Y_5_ (mg)	Seed yieldZ (kg/hm^2^)
2003	105	10	36	27	54	10	4
Total sample size(n)^b^		1050	3780	2835	5670	1050	420
2004	134	10	30	30	30	10	4
Total sample size(n)		1340	4020	4020	4020	1340	536
2005	60	10	30	30	30	10	4
Total sample size(n)		600	1800	1800	1800	600	240
2006	16	10	30	30	30	10	4
Total sample size(n)		160	480	480	480	160	64
Total n of 4 years(n)		**3150**	**10080**	**9135**	**11970**	**3150**	**1260**

a 100-seed was taken as one sample, at a seed water content of 7∼10%, then 10 of the 100-seed sample in each plot were averaged to obtain one sample of seed weight (Y_5_) of the plot; the total sample size (n) of Y_5_ = 10×105 = 1050 in 2003.

b Total sample size (n)  =  Sample size of plots (N) × Sample size of each plot (n), e.g., the number of spikelets fertile tiller^-1^ from 36 fertile tillers in each plot in 2003 was counted, then averaged as spikelets fertile tillers^-1^ (Y_2_) of the plot, so, the total sample size (n) of Y_2_ = 105×36 = 3780.

### Statistics and Analytical Method

Analyses of variance and Pearson correlation analyses were performed using the SAS Version 8.2 program [62]. The general linear model (PROC GLM) was used to assess the ridge model. Then, a Qbasic program was written for the path coefficient analysis; furthermore, Duncan's multiple range test for Z and Y_1_ to Y_5_ were performed. Data were transformed when necessary using logarithmic and power transformations in order to avoid the effects of highly inter-correlated, leading to multi-collinearity among Y_1_ to Y_5_ with Z.

To establish a reliable model, combined data for all of the Z and Y_1_ to Y_5_ in Visio FoxPro, totaling 315 samples of Z (105+134+60+16 = 315) with their corresponding components (Y_1_ to Y_5_) over the four years studied, were taken as the natural logarithm because, mathematically, they did not influence the essential relations of the variables [37,39,63].

If S  =  In Z, C*_i_* =  In Y*_i_*, (*i* =  1 to 5), then S and C_1_ to C_5_ were used for the ridge regression analyses [39], ridge regression model is:
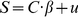
(6)


Where S is an *n*×1 vector of observations on a response variable, C is an *n*×*p* matrix of observations on *p* explanatory variables, ß is the *p*×1 vector of regression coefficients and ***u*** is an *n*×1 vector of residuals satisfying *E* (**u** ¯) =  **C** ˙, *E* (***uu***′)  = δ^2^ I. It is assumed that C and S have been scaled so that C′C and S′S are matrices of correlation coefficients [39]. Here *n*  =  315, *p* = 5. Thus,
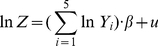
(7)


The above logarithmic model (7) was transformed to an exponential function as:
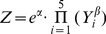
(8)


Where α, β are constants.

Formula (8) was used to estimate the Z of all 315 samples, and it was denoted as Z_estimated_; the actual seed yields were denoted as Z_actual_.

A general linear regression model was used to assess the Z_actual_, as compared to Z_estimated_, and an analysis of variance was used to assess the dependent variable Z_actual_ and the parameter estimates of Z_estimated_.

The linear regression model is:
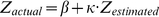
(9)


So, via formula (9), the model was adjusted to
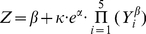
(10)


The separate analyses for the four years provided useful information. Simple statistics (PROC MEAN) was made on the results and ridge plots were did.

## Supporting Information

Figure S1 Monthly rainfall and mean temperature in Juquan, Guansu province, China in 2003, 2004, 2005 and 2006.
(TIF)

Table S1 Field Experimental design and factors in (*Psathyrostachys juncea* Nevski).
(DOC)

Table S2 Statistics of Y_1_∼Y_5_, Z (*Psathyrostachys juncea* Nevski) for year 2003 ∼ 2006.
(DOC)

Table S3 Dates of flowering and seed harvesting in 2003, 2004, 2005 and 2006.
(DOC)
