# Supplementary figures and images for: Path and Ridge Regression Analysis of Seed Yield and Seed Yield Components of Russian Wildrye (Psathyrostachys juncea Nevski) under Field Conditions
Source: PLoS One. 2011 Apr 18;6(4):e18245. doi: 10.1371/journal.pone.0018245 (PMC3078908; doi:10.1371/journal.pone.0018245)

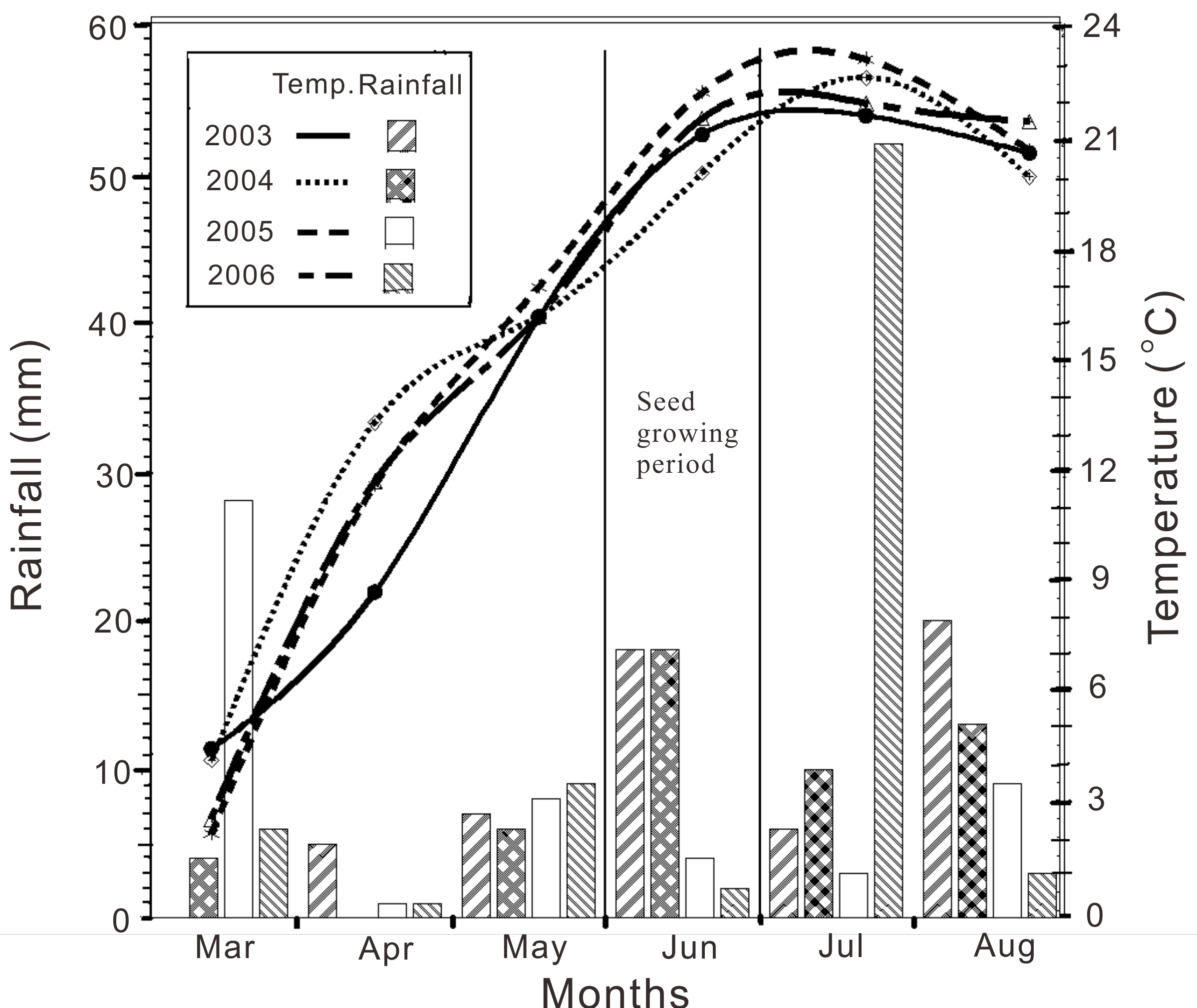

Supplement: Figure S1 — Monthly rainfall and mean temperature in Juquan, Guansu province, China in 2003, 2004, 2005 and 2006. (TIF) [file pone.0018245.s001.tif]
