# Supplementary material for: Path and Ridge Regression Analysis of Seed Yield and Seed Yield Components of Russian Wildrye (Psathyrostachys juncea Nevski) under Field Conditions
Source: PLoS One. 2011 Apr 18;6(4):e18245. doi: 10.1371/journal.pone.0018245 (PMC3078908; doi:10.1371/journal.pone.0018245)
Supplement: Table S1 — Field Experimental design and factors in (Psathyrostachys juncea Nevski). (DOC) [file pone.0018245.s002.doc]

**Table S1. Field Experimental design and factors in (***Psathyrostachys juncea* Nevski.)

| Field experimental design groups | Matrixes applied | Experimental factors | Repeat | Plots  (treatments) |
| --- | --- | --- | --- | --- |
| A. 2-D-optimum design(1)^a^ | 2-D-optimum matrix | 2 (X_3_，X_4_) | 3 | 18 |
| B. 2-D-optimum design(2)^a^ | 2-D-optimum matrix | 2 (X_3_，X_4_) | 1 | 6 |
| C. Quinque-factor orthogonal design | Compounding matrix | 5 (X_1_~X_5_) | 1 | 36 |
| D. Bin-factor orthogonal contract plots | Compounding matrix | 2 (X_2_,X_3_+X_4_) | 1 | 22 |
| E. Tri-factor orthogonal rotary design | Compounding matrix | 3 (X_1_,X_3_,X_6_) | 1 | 23 |
| F. Quinque-factor orthogonal design | L_8_(4^1^×2^4^) | 5 (X_1_,X_7_~X_10_) | 4 | 32 |
| CK | -- | -- | -- | 6 |
| total |  | 10(X_1_~X_10_) | -- | 143 |

^a^Applied N and P_2_O_5_ differently between design (1) and (2); X_1_~X_10_ orderly stand for: time of fertilizing, quantity of irrigation, applied nitrogen, applied P_2_O_5_, planted density, amount of spray plant regulator Paclobutrazol(PP333), irrigation time, density manipulation, time of cut post-harvest stubbles, and burning post-harvest stubbles, respectively.
