# Supplementary material for: Path and Ridge Regression Analysis of Seed Yield and Seed Yield Components of Russian Wildrye (Psathyrostachys juncea Nevski) under Field Conditions
Source: PLoS One. 2011 Apr 18;6(4):e18245. doi: 10.1371/journal.pone.0018245 (PMC3078908; doi:10.1371/journal.pone.0018245)
Supplement: Table S2 — Statistics of Y1∼Y5, Z (Psathyrostachys juncea Nevski) for year 2003 ∼ 2006. (DOC) [file pone.0018245.s003.doc]

**Table S2. Statistics of Y_1_~Y_5_、Z（*Psathyrostachys juncea* Nevski**.）for year 2003 ~ 2006

| Variable | year | N | Mean | Std-Dev | Std-Error | Minimum | Maximum | t-Value | Pr>|t| |
| --- | --- | --- | --- | --- | --- | --- | --- | --- | --- |
| Y1 | 2003 | 105 | 205.673 | 70.739 | 6.903 | 76.110 | 415.180 | 29.79 | <.0001 |
|  | 2004 | 134 | 542.307 | 262.220 | 22.652 | 56.000 | 1108.200 | 23.94 | <.0001 |
|  | 2005 | 60 | 178.089 | 86.247 | 11.134 | 46.926 | 404.932 | 15.99 | <.0001 |
|  | 2006 | 16 | 338.472 | 127.332 | 31.833 | 104.444 | 504.444 | 10.63 | <.0001 |
| Y2 | 2003 | 105 | 90.217 | 2.715 | 0.265 | 79.730 | 96.960 | 340.45 | <.0001 |
|  | 2004 | 134 | 89.542 | 8.432 | 0.728 | 73.360 | 105.760 | 122.92 | <.0001 |
|  | 2005 | 60 | 82.341 | 3.713 | 0.479 | 74.880 | 96.210 | 171.76 | <.0001 |
|  | 2006 | 16 | 81.135 | 4.884 | 1.221 | 73.800 | 93.420 | 66.45 | <.0001 |
| Y3 | 2003 | 105 | 4.590 | 0.754 | 0.074 | 3.010 | 6.250 | 62.36 | <.0001 |
|  | 2004 | 134 | 2.358 | 0.187 | 0.016 | 1.976 | 2.800 | 145.60 | <.0001 |
|  | 2005 | 60 | 2.293 | 0.221 | 0.029 | 1.653 | 2.630 | 80.33 | <.0001 |
|  | 2006 | 16 | 2.232 | 0.250 | 0.062 | 1.767 | 2.633 | 35.75 | <.0001 |
| Y4 | 2003 | 105 | 2.141 | 0.334 | 0.033 | 1.500 | 3.056 | 65.62 | <.0001 |
|  | 2004 | 134 | 2.054 | 0.182 | 0.016 | 1.660 | 2.590 | 130.47 | <.0001 |
|  | 2005 | 60 | 1.587 | 0.230 | 0.030 | 1.451 | 2.099 | 53.42 | <.0001 |
|  | 2006 | 16 | 1.749 | 0.220 | 0.055 | 1.429 | 2.133 | 31.74 | <.0001 |
| Y5 | 2003 | 105 | 3.461 | 0.184 | 0.018 | 2.933 | 3.834 | 192.83 | <.0001 |
|  | 2004 | 134 | 3.093 | 0.244 | 0.021 | 2.350 | 3.680 | 146.60 | <.0001 |
|  | 2005 | 60 | 3.387 | 0.535 | 0.069 | 2.262 | 4.055 | 49.05 | <.0001 |
|  | 2006 | 16 | 2.856 | 0.378 | 0.095 | 2.162 | 3.516 | 30.21 | <.0001 |
| Z | 2003 | 105 | 964.427 | 336.873 | 32.875 | 358.410 | 1969.650 | 29.34 | <.0001 |
|  | 2004 | 134 | 1483.82 | 689.823 | 59.592 | 73.644 | 2763.889 | 24.90 | <.0001 |
|  | 2005 | 60 | 541.329 | 267.231 | 34.500 | 154.036 | 1161.300 | 15.69 | <.0001 |
|  | 2006 | 16 | 714.442 | 226.883 | 56.721 | 198.434 | 989.761 | 12.60 | <.0001 |

Y_1_：Fertile tillers/m^2^； Y_2_：Spikelets/Fertile tillers； Y_3_：Florets/spikelet；Y_4_：Seed numbers/spikelet； Y_5_：Seed weight(mg)；Z：Seed yield(kg/hm^2^).
