# Supplementary material for: Path and Ridge Regression Analysis of Seed Yield and Seed Yield Components of Russian Wildrye (Psathyrostachys juncea Nevski) under Field Conditions
Source: PLoS One. 2011 Apr 18;6(4):e18245. doi: 10.1371/journal.pone.0018245 (PMC3078908; doi:10.1371/journal.pone.0018245)
Supplement: Table S3 — Dates of flowering and seed harvesting in 2003, 2004, 2005 and 2006. (DOC) [file pone.0018245.s004.doc]

**Table S3**. Dates of flowering and seed harvesting in 2003, 2004, 2005 and 2006.

|  | Year | | | |
| --- | --- | --- | --- | --- |
|  | 2003 | 2004 | 2005 | 2006 |
| Flowering date | 29 May | 31 May | 30 May | 31 May |
| Harvesting date | 2 July | 4 July | 29June | 1 July |
